# Hydromorphone Protects against CO_2_ Pneumoperitoneum-Induced Lung Injury via Heme Oxygenase-1-Regulated Mitochondrial Dynamics

**DOI:** 10.1155/2021/9034376

**Published:** 2021-04-09

**Authors:** Jia Shi, Shi-Han Du, Jian-Bo Yu, Yan-Fang Zhang, Si-Meng He, Shu-An Dong, Yuan Zhang, Li-Li Wu, Cui Li, Hai-Bo Li

**Affiliations:** ^1^Department of Anesthesiology and Critical Care Medicine, Tianjin Nankai Hospital, Tianjin Medical University, Tianjin 300100, China; ^2^Department of Anesthesiology and Critical Care Medicine, Tianjin Nankai Hospital, Nankai University, Tianjin 300100, China

## Abstract

Various pharmacological agents and protective methods have been shown to reverse pneumoperitoneum-related lung injury, but identifying the best strategy is challenging. Herein, we employed lung tissues and blood samples from C57BL/6 mice with pneumoperitoneum-induced lung injury and blood samples from patients who received laparoscopic gynecological surgery to investigate the therapeutic role of hydromorphone in pneumoperitoneum-induced lung injury along with the underlying mechanism. We found that pretreatment with hydromorphone alleviated lung injury in mice that underwent CO_2_ insufflation, decreased the levels of myeloperoxidase (MPO), total oxidant status (TOS), and oxidative stress index (OSI), and increased total antioxidant status (TAS). In addition, after pretreatment with hydromorphone, upregulated HO-1 protein expression, reduced mitochondrial DNA content, and improved mitochondrial morphology and dynamics were observed in mice subjected to pneumoperitoneum. Immunohistochemical staining also verified that hydromorphone could increase the expression of HO-1 in lung tissues in mice subjected to CO_2_ pneumoperitoneum. Notably, in mice treated with HO-1-siRNA, the protective effects of hydromorphone against pneumoperitoneum-induced lung injury were abolished, and hydromorphone did not have additional protective effects on mitochondria. Additionally, in clinical patients who received laparoscopic gynecological surgery, pretreatment with hydromorphone resulted in lower serum levels of club cell secretory protein-16 (CC-16) and intercellular adhesion molecule-1 (ICAM-1), a lower prooxidant-antioxidant balance (PAB), and higher heme oxygenase-1 (HO-1) activity than morphine pretreatment. Collectively, our results suggest that hydromorphone protects against CO_2_ pneumoperitoneum-induced lung injury via HO-1-regulated mitochondrial dynamics and may be a promising strategy to treat CO_2_ pneumoperitoneum-induced lung injury.

## 1. Introduction

The benefits of laparoscopic techniques include decreased blood loss, shorter hospital stays, and faster resumption of daily activities [1]. However, the establishment of pneumoperitoneum during laparoscopic surgery often has detrimental effects on respiratory function, and this is especially true for the patients in the Trendelenburg position during gynecologic surgery [2, 3]. Oxidative stress induced by excess reactive oxygen species (ROS) was identified as one of the mechanisms of CO_2_ pneumoperitoneum-induced lung injury [4]. The most likely mechanisms of oxidative stress resulting from CO_2_ pneumoperitoneum are tissue trauma-related inflammation, ischemia/reperfusion due to changes in abdominal pressure, and diaphragmatic dysfunction [5]. Excess ROS can damage cellular components, aggravate inflammatory responses, and cause cellular injury and organ dysfunction [6].

Mitochondria serve as key regulators of a host of cellular processes, sources of intracellular ROS, and targets of ROS-mediated damage [7]. Mitochondria undergo continual fusion and fission to maintain their integrity, structure, shape, and function when cells experience environmental stress [8]. Mitochondrial fusion is regulated by three GTPases, namely, mitofusins 1 and 2 (Mfn1 and Mfn2) and optic atrophy 1 (OPA1), whereas dynamin-related protein 1 (Drp1) and fission 1 (Fis1) are required for mitochondrial fission [9]. Mitochondrial dynamics are crucial for addressing high energy demands to maintain physiological cell functions. Impairment of mitochondrial dynamics related to excessive ROS production can trigger various pathologic processes, such as ischemic tissue injury, chronic lung disease, and diabetes, under conditions of cell stress [10–12]. A recent study suggested that intra-abdominal hypertension causes irreversible damage to mitochondria in the intestines of rabbits [13], unveiling an important pathogenic role of mitochondrial dynamics imbalance in pneumoperitoneum-related organ injury.

Heme oxygenase-1 (HO-1), a stress-responsive enzyme induced by ROS and cytokines that catalyzes the degradation of heme into carbon monoxide, biliverdin, and free iron, exerts beneficial antioxidant, anti-inflammatory, and cytoprotective effects [14]. Our previous studies confirmed that the HO-1 system exerts antioxidant effects to defend against endotoxin-induced lung injury by preserving mitochondrial dynamics [15, 16]. Therefore, HO-1-regulated mitochondrial dynamics may serve as a potential target for resisting oxidative and cellular stress. Given the limitations of HO-1 application in the clinic, identification of a common, safe, convenient drug that can regulate HO-1 may be beneficial [17].

Hydromorphone, a semisynthetic derivative of morphine, is widely used for acute and chronic pain management in the USA and European countries and has a quicker onset and better analgesic efficacy than morphine [18]. It has been demonstrated that some classic opioids (such as morphine) protect the heart from ischemia/reperfusion injury by inhibiting mitochondrial oxidative stress [19]. Moreover, hydromorphone shows distinct antioxidative stress effects by reducing the levels of ROS, thus protecting animal models of ischemic cerebral injury [20]. These findings strongly support our hypothesis that hydromorphone may attenuate CO_2_ pneumoperitoneum-induced lung injury and that HO-1-mediated mitochondrial dynamics may be involved in this process.

To test our hypothesis, the current research used a C57BL/6 mouse model of pneumoperitoneum and enrolled clinical patients who received elective laparoscopic gynecological surgery. We revealed that hydromorphone targets the balance of HO-1-mediated mitochondrial dynamics to alleviate CO_2_ pneumoperitoneum-induced lung injury.

## 2. Materials and Methods

### 2.1. Human Research

#### 2.1.1. Human Patients

In this study, fifty patients who received elective laparoscopic gynecological surgery at the Department of Anesthesiology and Critical Care Medicine of Tianjin Medical University Nankai Hospital in China from July 2017 to August 2018 were enrolled. All procedures were conducted in compliance with protocols approved by the Ethics Committee of Tianjin Medical University Nankai Hospital in China (no. 2017-007P). Informed consent was obtained from participants or their families. The study protocol was registered at https://chictr.org.cn (ChiCTR-IOR-17011733).

Blood samples were collected from fifty patients with an ASA physical status of I or II who received elective laparoscopic gynecological surgery and pretreatment with hydromorphone (*n* = 25) or morphine (*n* = 25) under general anesthesia (aged 25-65 y, body weight of 40-70 kg). All patients included in the study had no history of chronic pain, psychosis, or long-term use of analgesic drugs. The exclusion criteria included a history of the use of drugs such as opioids/NSAIDs, phenothiazines, sedative-hypnotic drugs, MAO inhibitors, tricyclic antidepressants, and antihistamines; asthma; mental illness; severe cardiopulmonary disease; and severe liver and kidney diseases. Group H received slow intravenous infusion of 2 mg hydromorphone (dose: 2 mg/ml, Yichang Humanwell Pharmaceutical Co., Ltd., NMPN: H20120100; lot number: TD2012-0010), and group S received slow intravenous infusion of 10 mg morphine (dose: 1 ml/10 mg, Northeast Pharmaceutical Group Shenyang First Pharmaceutical Co., Ltd., NMPN: H21022436; lot number: 180115-1) 15 min before skin incision. Then, all patients received sevoflurane inhalation anesthesia. The initial sevoflurane inhalation concentration was set at 8%, the fresh gas flow of oxygen was set at 5 l/min, and 0.15 mg/kg *cis*-atracurium was administered when the patients had a BIS < 65. Tracheal intubation was performed when the patient's muscles were relaxed and the BIS was less than 40 to allow mechanical ventilation with an anesthesia machine (FiO_2_: 100%; Vt: 6-8 ml/kg; RR: 10-12/min). Pneumoperitoneum was established by insufflation of carbon dioxide gas through the laparoscopic pneumoperitoneum needle into the abdominal cavity, and the intra-abdominal pressure was maintained at less than 12 mmHg. Anesthesia was maintained with 3%-4% sevoflurane, a MAC of 1.2~1.4, an oxygen flow rate of 1.5-2 l/min, and a PetCO_2_ of 35-45 mmHg. *cis*-Atracurium was injected discontinuously to maintain muscle relaxation, and the BIS was maintained at 45-55 during the surgery (2 h ≤ duration ≤ 3 h).

#### 2.1.2. Collection of Human Samples

Venous blood samples were taken when the patient entered the operating room (T0); immediately before pneumoperitoneum (T1); 30 min (T2), 1 h (T3), and 2 h (T4) after establishment of pneumoperitoneum; and 5 min after tracheal extubation (T5). To rule out interference from the complement proteins, blood samples were collected in EDTA-containing tubes. The samples were then centrifuged at 1500 × *g* to 2000 × *g* at 4°C for 15 min to obtain the serum, and the supernatant was collected and stored at -80°C. Changes in intercellular adhesion molecule-1 (ICAM-1) and club cell secretory protein-16 (CC-16) levels in the blood samples were measured by enzyme-linked immunosorbent assay (ELISA) kits according to the manufacturer's protocol (R&D Systems, Minneapolis, MN, USA). A 3,3′,5,5-tetramethylbenzidine-based prooxidant-antioxidant balance (PAB) assay, in which the cation of 3,3′,5,5-tetramethylbenzidine was used as a redox indicator involved in two different types of reactions, was performed according to previous studies [21]. As described by Camhi et al. [22] with slight modifications, the level of bilirubin, which was determined with a UV spectrophotometer, was used to assess HO-1 activity.

### 2.2. Animal Experiments

#### 2.2.1. Animals

Male C57BL/6 mice aged 6-8 weeks and weighed 18~20 g were housed at a constant temperature (23-25°C) on a 12 h dark/12 h light cycle and provided free access to standard mouse food and water. Food was withheld 12 h before anesthesia, but free access to water was allowed. All animal experiments were performed in accordance with NIH guidelines for the care and use of laboratory animals and approved by the Animal Ethical and Welfare Committee (AEWC) of the Institute of Radiation Medicine Chinese Academy of Medical Sciences (no. IRM-DWLL-2017034).

#### 2.2.2. Establishment of a Mouse CO_2_ Pneumoperitoneum-Induced Lung Injury Model

Mice were weighed and anesthetized by an intraperitoneal injection of 1% pentobarbital sodium at a dose of 40 mg/kg. Microrenathane catheters (MRE-025, Braintree Scientific, Braintree, MA) were inserted into the femoral artery of each mouse after stable anesthesia was achieved [23]. Then, the arterial catheters were connected to sensors to monitor the femoral artery mean arterial pressure. According to the results of our preexperiments (Supplementary Figure S1), a total of 120 *μ*g (10 *μ*l) hydromorphone (group HR) or 0.9% NS (10 *μ*l) (group P) was slowly injected intraperitoneally into the mice 15 min before the establishment of pneumoperitoneum. Then, pneumoperitoneum was established by inserting a Veress needle into the peritoneal cavity and insufflating CO_2_ through the Wisap CO_2_ gas insufflator (Wisap, Sauerlach, Germany). The insufflator was used to set the intra-abdominal pressure to 15 mmHg for insufflation (for 1 h) and deflation with CO_2_ (for 3 h). Moderate abdominal distention was observed in mice subjected to CO_2_ insufflation, indicating that the model was made established. During the procedure, anesthesia was maintained by i.p. injection of 1% pentobarbital sodium. Pneumoperitoneum was not established in the control group (group C).

#### 2.2.3. Measurement of Myeloperoxidase (MPO) Activity in the Serum

Mouse blood samples were taken from the retroorbital plexus under anesthesia and collected in tubes. After collection, the blood was left undisturbed at room temperature for 30 min to allow it to clot. Serum was obtained by centrifugation at 2000 × *g* for 10 min, and then, samples were stored at -20°C. MPO activity was determined using a test kit (#A044, Nanjing Jiancheng Bioengineering Institute, CN) according to the manufacturer's instructions. Briefly, the serum samples were diluted with equal amounts of extraction buffer, and reaction buffer was added. After incubation at 37°C, the reaction was stopped with stop solution, and the absorbance of the colorimetric reaction was measured by using a spectrophotometer at 460 nm (Lambda 35, PerkinElmer, USA). The results are presented in U/l.

#### 2.2.4. Determination of TAS and TOS in the Serum

Total antioxidant status (TAS) and total oxidant status (TOS) were determined by kits (#KC5200/#KC5100, Immundiagnostik, Germany) according to the spectrophotometric methods developed by Erel [24]. The results are presented in *μ*mol/l. An indicator of the degree of oxidative stress, the oxidative stress index (OSI), was calculated using the following formula: OSI = (TOS/TAS) × 100 (arbitrary units).

#### 2.2.5. Histological Analysis of Mice

After blood sampling, the mice were sacrificed by cervical dislocation under deep anesthesia, and their lungs were rapidly removed. The right lungs were fixed with 10% formaldehyde and routinely processed to generate paraffin sections (4 *μ*m) for standard H&E staining. Ten different fields of each slice were examined under a light microscope (×200) by a blinded pathologist. Histological scores were assigned for each of the following pathologic features to evaluate the degree of injury in lung tissues: intra-alveolar hemorrhage, congestion, thickness of the alveolar wall, and inflammation infiltration or aggregation in air space or vessel wall. Each parameter was graded on a scale from 0 to 5 as follows: 0, minimal; 1, mild; 2, moderate; 3, severe; and 4, maximal [25].

#### 2.2.6. Determination of Mitochondrial DNA Copy Number by Real-Time PCR

Total DNA was isolated from right lung tissues (50 mg) by using the DNeasy Blood and Tissue Kit (#DN10, Aidlab Biotechnologies, CN). The relative mtDNA copy number was measured by PCR with SYBR-green PCR master mix (#Q511-02, Vazyme Biotech, CN) using the relative standard curve method. mtDNA content was determined by amplification of the 117 bp mtDNA fragment. Relative quantification of mtDNA content was determined based on the ratio of the 117 bp mtDNA fragment to the nuclear-encoded *β*-actin. Primers for genes were designed using NCBI Primer-BLAST (http://www.ncbi.nlm.nih.gov/tools/primer-blast/). Product specificity and reaction efficiencies were verified for each primer pair. The primer sequences for 117 bp mtDNA fragment were 5′-CCCAGCTACTACCATCATTCAAGT-3′ (forward) and 5′-GATGGTTTGGGAGATTGGTTGATG-3′ (reverse). The primer sequences for nuclear-encoded *β*-actin were 5′-GTACCACCATGTACCCAGGC-3′ (forward) and 5′-GCAGCTCAGTAACAGTCCGC-3′ (reverse). The mitochondrial to nuclear DNA (mt/nDNA) ratio was calculated using the 2^-*ΔΔ*CT^ method to assess the relative mtDNA copy number.

#### 2.2.7. Analysis of Mitochondrial Structure by Electron Microscopy

Small pieces of right lung tissue were fixed in 2.5% glutaraldehyde at 4°C overnight, postfixed with 1% osmium tetroxide for 2 h, and washed three times with PBS. The tissues were dehydrated in a gradient series of ethanol and then incubated overnight in 100% ethanol. All tissue samples were sectioned at 70 nm and prior to staining with uranyl acetate and lead citrate. Then, the samples were visualized under a transmission electron microscope (TEM, Tecnai G2, FEI).

#### 2.2.8. Immunofluorescence Staining for HO-1 in Lung Tissues

Paraffin sections of right lung tissues were washed three times with PBS. After fixation with 4% paraformaldehyde (PFA) and 0.3% Triton X-100 for 20 min, the tissue sections were incubated with an anti-HO-1 (1 : 100) (Abcam, USA) primary antibody at 4°C overnight. The next day, the slides were incubated with a FITC-conjugated anti-mouse secondary antibody (1 : 100) (Jackson ImmunoResearch, USA) for 1 h at 25°C. The nuclei were counterstained with 4′,6-diamidino-2-phenylindole (DAPI). The sections were observed by using a fluorescence microscope (#BX53, OLYMPUS, Japan).

#### 2.2.9. Administration of Small-Interfering RNAs (siRNAs) to Mice

siRNA against mouse HO-1 was synthesized by Santa Cruz Biotech (Santa, USA). Entranster™ in vivo transfection reagent was prepared according to the manufacturer's instructions (Engreen Biosystem, CN). HO-1-siRNA (#SC-35555, Santa, USA) or NC-siRNA (#siN0000002-1-10, RiboBio, CN) was injected into the mice via the tail vein 48 h before experimental intervention.

#### 2.2.10. TUNEL Assay

Paraffin-embedded lung tissue sections were stained with a TUNEL detection kit (Roche Applied Science) according to the manufacturer's instructions. The slides were observed with a fluorescence microscope.

#### 2.2.11. Bronchoalveolar Lavage (BAL) and Preparation of Bronchoalveolar Lavage Fluid (BALF)

BAL was performed three times through a tracheal cannula. PBS was used to lavage both lungs, and 80% of the total lavage volume was recovered. BALF was collected and separated by centrifugation at 1000 × *g* for 10 min (4°C), and the supernatant was stored at -80°C for further analysis.

#### 2.2.12. Lactate Dehydrogenase (LDH) Assay

LDH activity in the BALF following different treatments was measured by a microplate reader (#RT-6100, Rayto Life and Analytical Sciences Co. Ltd., China) according to the manufacturer's instructions (Nanjing Jiancheng Bioengineering Institute Co. Ltd., Nanjing, China).

#### 2.2.13. Determination of Mitochondrial Swelling

Intact mitochondria isolated from pulmonary samples were suspended in swelling buffer. Mitochondrial swelling was assessed in all groups by measuring the change in the absorbance of the lung mitochondrial suspension at 540 nm using a microplate reader (#RT-6100, Rayto Life and Analytical Sciences Co. Ltd., China). The reaction conditions were set at 25°C. A decrease in absorbance indicated mitochondrial swelling [26, 27].

#### 2.2.14. Western Blot Analysis

Mitochondria proteins were isolated from the right lung tissues of mice using a mitochondrial protein fraction kit (Beyotime, Shanghai, China) according to the manufacturer's protocols. Protein concentrations were determined by using a Pierce BCA Protein Assay Kit (Aidlab Biotechnologies, Beijing, China). Equal amounts of proteins were separated on a 12% SDS-PAGE gel and then transferred to a PVDF membrane (Millipore, USA). After blocking with 5% fat-free milk for 1 h, the membrane was incubated with a rabbit anti-HO-1 antibody (1 : 2000, #10701, PTG, USA), mouse anti-MFN1 antibody (1 : 1000, #ab57602, Abcam, UK), rabbit anti-MFN2 antibody (1 : 1000, #12186, PTG, USA), rabbit anti-OPA1 antibody (1 : 1000, #ab157457, Abcam, UK), rabbit anti-DRP1 antibody (1 : 1000, #ab184247, Abcam, UK), rabbit anti-FIS1 antibody (1 : 1000, #ab71498, Abcam, UK), and mouse anti-*β*-actin antibody (1 : 8000, #KM9001, Tianjin Sungene Biotech, CN) at 4°C overnight. After being washed four times with TBST, the membrane was incubated with horseradish peroxidase- (HRP-) conjugated secondary antibodies (1 : 3000, #E030130, Earthox, USA) at room temperature for 1 h. The protein bands were visualized by enhanced chemiluminescence (#3300 Mini, Clinx Science Instruments, CN), and the relative densities of the bands were quantified by using the ImageJ software.

### 2.3. Statistical Analysis

For mouse experiments, the data are presented as the mean ± SD, except for lung injury scores, which are presented as box plots. One-way ANOVA followed by Bonferroni correction was used for analyzing differences between multiple groups. Human data are presented as the mean ± standard deviation (SD) or median (25th percentile, 75th percentile) as indicated. Comparisons between two groups were made using *t*-test, the Mann–Whitney *U* test, or Fisher's exact test as appropriate. Comparisons between multiple groups were performed by two-way repeated-measures ANOVA. All results were analyzed using GraphPad Prism 8 software (GraphPad Software, Inc., San Diego, CA, USA) and Statistical Program for Social Sciences 20.0 software (SPSS, Inc., Chicago, IL, USA). A *P* value < 0.05 was considered significant. Randomization was used to assign all animals and patients to treatment groups. All researchers were blinded to the grouping information and the data being collected.

## 3. Results

### 3.1. Hydromorphone Alleviates Lung Injury Induced by CO_2_ Pneumoperitoneum in Mice

**Figure 1:**
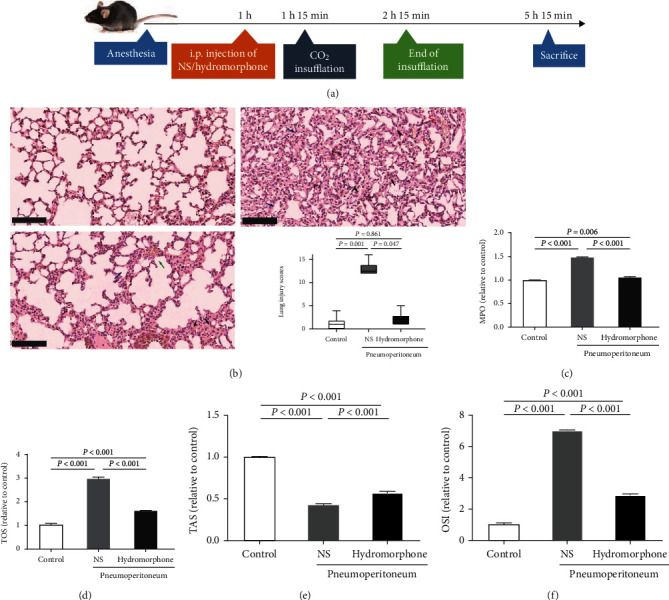
Hydromorphone alleviates pneumoperitoneum-induced lung injury in mice. (a) Schematic diagram depicting the animal treatment procedure. C57BL/6 mice were treated with 120 *μ*g hydromorphone or 10 *μ*l 0.9% NS i.p. 1 h after pentobarbital sodium anesthesia. Then, CO_2_ insufflation was performed with 15 min after hydromorphone administration. Pneumoperitoneum was maintained for 1 h, and the peritoneal gas was then desufflated. The mice were sacrificed 3 h after the end of pneumoperitoneum. (b) Representative H&E-stained lung tissues of mice. Red arrow indicated intra-alveolar hemorrhage, black arrows indicated inflammatory cell infiltration, green arrow indicated intra-alveolar congestion, and blue arrows indicated thickening of the alveolar wall. Original magnification, ×200, scale bar: 100 *μ*m. Lung injury scores are shown in box plots (25th to 75th percentiles; the horizontal lines represent the median, maximum, and minimum values). *P* values were calculated by the independent-samples Kruskal-Wallis test. (c) Relative myeloperoxidase (MPO) activity in the sera of mice in different treatment groups. The data are the mean ± SD. *P* values were calculated by one-way ANOVA followed by Bonferroni correction. (d–f) The relative total oxidant status (TOS), total antioxidant status (TAS), and oxidative stress index (OSI). The data are the mean ± SD. *P* values were calculated by one-way ANOVA followed by Bonferroni correction (*n* = 3 to 6 per group).

To investigate the effects of hydromorphone on CO_2_ pneumoperitoneum-induced lung injury, we developed a mouse model of pneumoperitoneum. C57BL/6 mice were given hydromorphone or normal saline (NS) 15 min before establishment of pneumoperitoneum, and serum samples and lung tissues were collected 4 h after modeling (Figure 1(a)). Under a light microscope, the lung slices of NS-pretreated mice exposed to pneumoperitoneum exhibited diffuse intra-alveolar hemorrhage, a thickened septum, dense alveolar congestion, and leukocyte infiltration. As expected, hydromorphone had beneficial effects on the architecture of the alveoli, alveolar congestion, and leukocyte infiltration in lung tissues. Quantitatively, the lung injury scores were significantly lower in hydromorphone-pretreated mice than in saline-pretreated mice (Figure 1(b)). In addition, hydromorphone protected against inflammation, as shown by reduced serum myeloperoxidase (MPO) activity (Figure 1(c)). Similarly, total oxidant status (TOS) in the serum was significantly higher in NS-pretreated mice than in hydromorphone-pretreated mice, while total antioxidant status (TAS) was lower in mice pretreated with NS. Consequently, the oxidative stress index (OSI) was significantly higher in NS-pretreated mice than in hydromorphone-pretreated mice (Figures 1(d)–1(f)).

### 3.2. Hydromorphone Improves Mitochondrial Morphology and Function following Pneumoperitoneum-Induced Lung Injury in Mice

**Figure 2:**
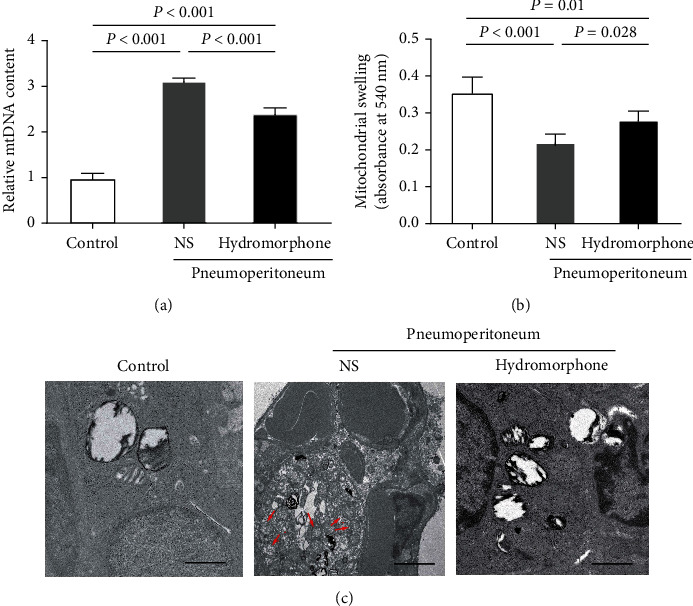
Hydromorphone ameliorates changes in mitochondrial morphology and mitochondrial function following pneumoperitoneum-induced lung injury. (a) Relative mtDNA content (*n* = 3 per group). Real-time PCR was used to quantify absolute mtDNA and nDNA levels. mtDNA expression was normalized to nDNA expression in each tissue sample. The data are the mean ± SD. *P* values were calculated by one-way ANOVA followed by Bonferroni correction. (b) Pneumoperitoneum-induced mitochondrial swelling and the effect of hydromorphone administration. The data are the mean ± SD. *P* values were calculated by one-way ANOVA followed by Bonferroni correction (*n* = 6 per group). (c) Representative transmission electron micrographs showing the ultrastructure of mitochondria in lung tissues (*n* = 3 per group). The red arrows indicated swollen, vacuolar, and fragmented mitochondria. Scale bars: 1 *μ*m.

To investigate the mechanism underlying the effect of hydromorphone on mitochondrial function in the mouse model of pneumoperitoneum-induced lung injury, the mitochondrial DNA (mtDNA) content in the lung tissues of the mice was determined. Pretreatment with hydromorphone decreased mtDNA content after induction of pneumoperitoneum (Figure 2(a)). Moreover, mitochondrial swelling (an indicator of impaired mitochondrial integrity) was increased in pulmonary mitochondria isolated from mice subjected to pneumoperitoneum and was attenuated by treatment with hydromorphone (Figure 2(b)). Electron microscopy showed abnormalities in mitochondrial ultrastructure, mainly mitochondrial swelling, vacuolization, and blurriness or disruption of mitochondrial cristae, in mice after induction of pneumoperitoneum. Smaller and more numerous and fragmented mitochondria were observed in mice subjected to pneumoperitoneum than in normal mice (see Figure 2(c), red arrows). In comparison, the structural alterations caused by pneumoperitoneum were attenuated when hydromorphone was applied prior to pneumoperitoneum exposure (Figure 2(c)).

### 3.3. Hydromorphone Upregulates HO-1 Expression and Preserves Mitochondrial Dynamics following Pneumoperitoneum-Induced Lung Injury in Mice

**Figure 3:**
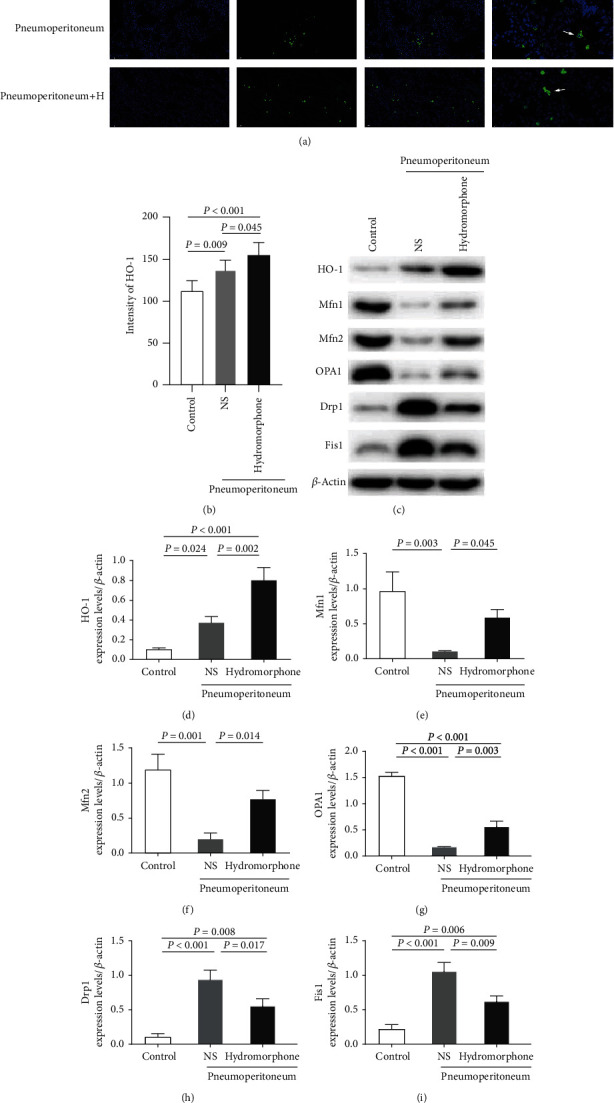
Hydromorphone upregulates HO-1 expression and preserves mitochondrial dynamics following pneumoperitoneum-induced lung injury in mice. (a) Immunofluorescence staining for HO-1 (green) in lung tissue sections from each group. The nuclei were stained blue. White arrows indicated positive staining areas of HO-1. Scale bar = 50 *μ*m. (b) The fluorescence intensity of HO-1. The data are the mean ± SD. *P* values were calculated by independent-samples *t*-test (*n* = 6 per group). (c) Representative immunoblots of (d) HO-1, (e) Mfn1, (f) Mfn2, (g) OPA1, (h) Drp1, and (i) Fis1 in lung tissues. The data are the mean ± SD. All *P* values were calculated by one-way ANOVA followed by Bonferroni correction (*n* = 3 per group).

Impairment of the balance between mitochondrial fusion and fission can disrupt the functional integrity of mitochondria and predispose the cell to apoptosis in the context of heart, lung, and kidney injury [15, 28, 29]. Therefore, we examined the effects of hydromorphone on the expression of HO-1 and several mitochondrial fusion (Mfn1/2 and OPA1) and fission (Drp1 and Fis1) proteins. As shown in Figures 3(a) and 3(b), immunofluorescence staining of lung sections showed that the expression of HO-1 increased in mice after induction of pneumoperitoneum and that hydromorphone further increased HO-1 expression in lung tissues. Furthermore, the expression of HO-1 and the mitochondrial fission proteins Drp1 and Fis1 was increased in mice after induction of pneumoperitoneum, whereas the expression of the mitochondrial fusion proteins Mfn1, Mfn2, and OPA1 was decreased. Furthermore, the levels of HO-1 and the mitochondrial fusion proteins Mfn1, Mfn2, and OPA1 in hydromorphone-pretreated mice were significantly higher than those in NS-pretreated mice, and the expression of the mitochondrial fission proteins Drp1 and Fis1 was suppressed in mice pretreated with hydromorphone (Figures 3(c)–3(i)). These findings suggested that hydromorphone might regulate mitochondrial dynamics and preserve mitochondrial function by upregulating HO-1 expression.

### 3.4. The Protective Effects of Hydromorphone against Pneumoperitoneum-Induced Lung Injury Were Abolished in Mice Transfected with HO-1-siRNA

**Figure 4:**
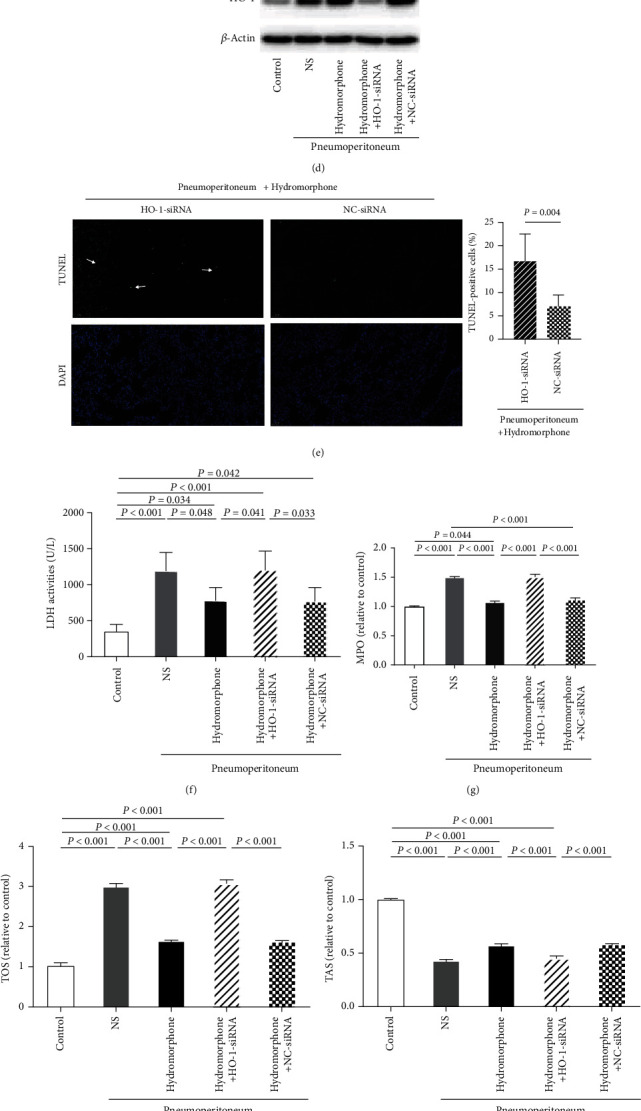
The protective effects of hydromorphone on pneumoperitoneum-induced lung injury were abolished in mice transfected with HO-1-siRNA. (a) The in vivo transfection reagent and HO-1-siRNA or NC-siRNA were injected into C57BL/6 mice via the tail vein 48 h before experimental intervention. NS/hydromorphone was injected after stable blood pressure was achieved in anesthetized mice. CO_2_ insufflation was then performed to induce lung injury in mice 15 min after NS/hydromorphone administration. (b) Immunofluorescence staining for and (c) the fluorescence intensity of HO-1 in the lung tissues of mice. The data are the mean ± SD. *P* values were calculated by independent-samples *t*-test (*n* = 6 per group). White arrows indicated positive staining areas of HO-1. Scale bar = 50 *μ*m. (d) The expression levels of HO-1 were measured by western blot analysis (*n* = 3 per group). (e) Representative images of TUNEL staining and measurements of TUNEL-positive cells in lung sections. The data are the mean ± SD. *P* values were calculated by independent-samples *t*-test (*n* = 6 per group). White arrows indicated TUNEL-positive cells. Scale bar: 50 *μ*m. (f) LDH activity in BALF from each group. The data are the mean ± SD. *P* values are calculated by one-way ANOVA followed by Bonferroni correction (*n* = 5 per group). (g) Serum myeloperoxidase (MPO) activity (*n* = 3 per group). The data are the mean ± SD. *P* values were calculated by one-way ANOVA followed by Bonferroni correction. (h–j) Relative total oxidative status (TOS), total antioxidant status (TAS), and oxidative stress index (OSI) (*n* = 3 per group). The data are the mean ± SD. All *P* values were calculated by one-way ANOVA followed by Bonferroni correction.

To further clarify whether hydromorphone exerted protective effects against lung injury induced by pneumoperitoneum through HO-1-mediated mitochondrial dynamics, we injected NC-siRNA or HO-1-siRNA into mice via the tail vein 48 h before surgery (Figure 4(a)). As shown in Figures 4(b)–4(d), immunofluorescence and western blotting suggested that the expression of HO-1 in lung tissues was successfully altered after transfection. Following pneumoperitoneum, pulmonary apoptosis was more pronounced in mice transfected with HO-1-siRNA than in those transfected with NC-siRNA even after pretreatment with hydromorphone, as shown by TUNEL staining (Figure 4(e) and Supplementary Figure S4). Furthermore, to quantitatively evaluate cytotoxicity, we measured LDH activity in BALF as a global marker of lung cell injury [30, 31]. As shown in Figure 4(f), LDH activity in BALF was significantly elevated in mice subjected to pneumoperitoneum, reflecting damage to the cell membrane. However, hydromorphone administration effectively prevented these alterations, and the protective effects of hydromorphone against pneumoperitoneum-induced cell injury were abolished in mice transfected with HO-1-siRNA. Consistently, hydromorphone did not improve the MPO activity, TOS, TAS, or the OSI in the serum in HO-1-siRNA-transfected mice (Figures 4(g)–4(j)).

**Figure 5:**
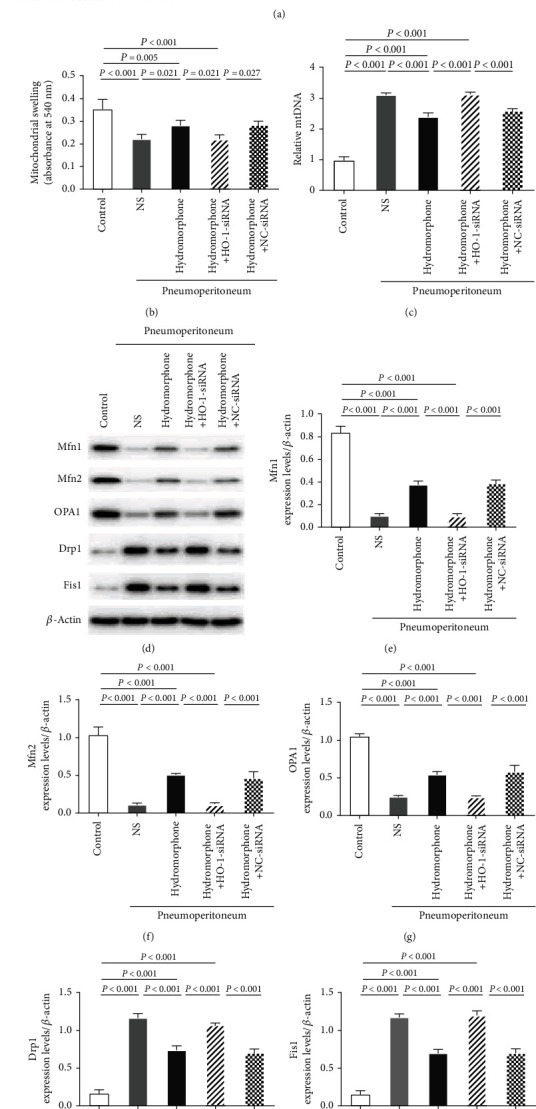
Hydromorphone protects against CO_2_ pneumoperitoneum-induced lung injury via HO-1-mediated mitochondrial dynamics. (a) Representative transmission electron micrographs showing the ultrastructure of mitochondria in lung tissues (*n* = 3 per group). The red arrows indicated swollen, vacuolar, and fragmented mitochondria. Scale bars: 2 *μ*m. (b) Mitochondrial swelling in pulmonary mitochondria isolated from experimental mice. The data are the mean ± SD. *P* values were calculated by one-way ANOVA followed by Bonferroni correction (*n* = 6 per group). (c) Relative mtDNA content (*n* = 3 per group). The data are the mean ± SD. *P* values are calculated by one-way ANOVA followed by Bonferroni correction. (d) Representative immunoblots of (e) Mfn1, (f) Mfn2, (g) OPA1, (h) Drp1, and (i) Fis1 in lung tissues (*n* = 3 per group). The data are the mean ± SD. All *P* values were calculated by one-way ANOVA followed by Bonferroni correction.

Notably, at the ultrastructural level, hydromorphone suppressed mitochondrial swelling, vacuolization, and mitochondrial fragmentation after induction of pneumoperitoneum in mice transfected with NC-siRNA but not in mice transfected with HO-1-siRNA (Figures 5(a) and 5(b)). Moreover, hydromorphone decreased the mtDNA content in mice transfected with NC-siRNA but not in mice transfected with HO-1-siRNA (Figure 5(c)). As expected, after induction of pneumoperitoneum, hydromorphone had no effects on mitochondrial dynamics in mice transfected with HO-1-siRNA (Figures 5(d)–5(i)). Taken together, these findings showed that HO-1 deficiency inhibits the protective effects of hydromorphone on mitochondrial dynamics in the context of pneumoperitoneum-induced lung injury.

### 3.5. Hydromorphone Ameliorates the CO_2_ Pneumoperitoneum-Induced Inflammatory Response and Oxidative Stress in Humans

To determine the clinical relevance of our findings, we collected blood samples from 50 patients who received elective laparoscopic gynecological surgery under general anesthesia (2 h ≤ duration ≤ 3 h) at six time points (T0 (when the patient entered the operating room), T1 (immediately before pneumoperitoneum), T2 (30 minutes after establishment of pneumoperitoneum), T3 (1 h after establishment of pneumoperitoneum), T4 (2 h after establishment of pneumoperitoneum), and T5 (5 min after tracheal extubation)). The patients were randomly assigned to groups and intravenously infused with 2 mg hydromorphone 15 min before skin incision (group H) or 10 mg morphine 15 min before skin incision (group S). There were no significant differences in baseline characteristics or surgical data between the two groups (Table 1).

Categorical variables are presented as *n* (%); continuous variables are reported as the median (25th percentile, 75th percentile).

**Figure 6:**
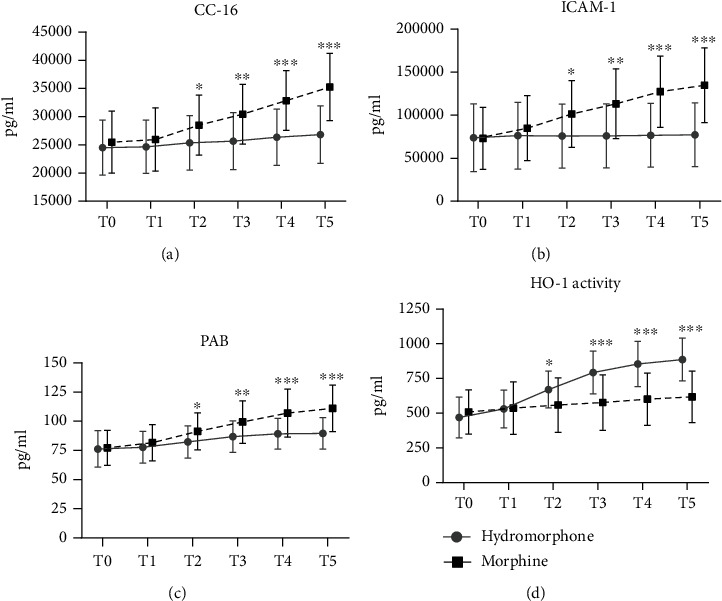
Hydromorphone ameliorates the CO_2_ pneumoperitoneum-induced inflammatory response and oxidative stress in humans. (a) CC-16 levels, (b) ICAM-1 levels, (c) the PAB, and (d) HO-1 activity in human blood samples from the two groups at five time points (*n* = 25 per group). The data are the mean ± SD. All *P* values were calculated by two-way repeated-measures ANOVA. ^∗^*P* < 0.05, ^∗∗^*P* < 0.01, and ^∗∗∗^*P* < 0.001 (the hydromorphone group vs. the morphine group). T0: when the patient entered the operating room; T1: immediately before pneumoperitoneum; T2: 30 minutes after establishment of pneumoperitoneum; T3: 1 h after establishment of pneumoperitoneum; T4: 2 h after establishment of pneumoperitoneum; T5: 5 min after tracheal extubation.

We measured the levels of club cell secretory protein-16 (CC-16) in the serum, which was proposed as a biomarker of injury in various lung diseases in recent clinical studies [32, 33]. The levels of CC-16 increased with the duration of surgery in both groups. Moreover, the level of CC-16 increased to a greater degree in patients pretreated with morphine than in patients pretreated with hydromorphone (Figure 6(a)). As CO_2_ pneumoperitoneum-induced lung injury is associated with systemic inflammatory and oxidative responses [34], we assessed the effects of hydromorphone on intercellular adhesion molecule-1 (ICAM-1) levels and the prooxidant-antioxidant balance (PAB), which has been shown to be closely related to inflammatory responses [35, 36]. As shown in Figures 6(b) and 6(c), ICAM-1 levels and the PAB in blood samples gradually increased with the duration of surgery in both groups. Compared with morphine, hydromorphone more obviously protected against inflammatory and oxidative responses, as shown by lower levels of ICAM-1 (Figure 6(b)) and a lower PAB (Figure 6(c)). Furthermore, the activity of the stress-responsive enzyme HO-1 was measured in the two groups. Unlike ICAM-1 levels and the PAB, HO-1 activity increased to a greater degree in patients pretreated with hydromorphone than in patients pretreated with morphine (Figure 6(d)).

## 4. Discussion

The present study showed that hydromorphone exerted antioxidative and anti-inflammatory effects to protect against CO_2_ pneumoperitoneum-induced lung injury in mice, and the protective mechanism probably depended on its capacity to upregulate HO-1 expression and preserve the mitochondrial dynamic equilibrium. Furthermore, hydromorphone could improve inflammatory and oxidative stress biomarkers in female patients who received gynecological laparoscopic surgery.

The lung is one of the most susceptible organs to high intra-abdominal pressure (IAP) caused by pneumoperitoneum. Insufflation of CO_2_ into the abdominal cavity elevates the diaphragm, increases the intrathoracic pressure, and decreases pulmonary blood flow, which results in oxidative stress injury in lung tissues [2, 3]. Deflation of pneumoperitoneum restores the pulmonary blood circulation to normal levels but generates free radicals and causes reperfusion injury, which exacerbates the pulmonary inflammatory response and oxidative stress [37]. Many strategies, such as reducing intra-abdominal pressure and administering pharmacological agents with anti-inflammatory and antioxidant effects, have been developed to decrease the severity of pneumoperitoneum-associated lung injury by enhancing the body's autodefense mechanisms [38, 39]. Some studies have shown that the use of opioids, such as morphine has many beneficial effects. Fukada and colleagues found that pretreatment with morphine ameliorates LPS-induced lung injury, suppresses lethal endotoxic shock, and improves survival rates in mice [40]. Hydromorphone, a semisynthetic morphine derivative, is 5-10 times more potent than morphine [41]. In addition to exerting analgesic effects, it has been shown that pretreatment with hydromorphone significantly reduces the levels of ROS in mouse glial cells under ischemic conditions and that the *μ*, *δ*, *κ* opioid receptors participate in the antioxidative effects of hydromorphone on glial cells [20]. Additionally, when used in postoperative pain management, hydromorphone had better anti-inflammatory effects than other sufentanil in patients who received thoracic surgery, as shown by lower levels of C-reactive protein (CRP) in the plasma and fewer pulmonary complications [42]. To investigate the role of hydromorphone in clinical patients receiving gynecological laparoscopic surgery, we used a dosage of hydromorphone equianalgesic to the dosage of morphine used in other studies [43]. CC-16 is regarded as a lung secretory protein that protects against oxidative stress and inflammation. During lung inflammation, CC-16 diffuses passively across the bronchoalveolar/blood barrier into peripheral blood [44]. Thus, serum CC-16 has been applied as a sensitive peripheral blood biomarker of lung injury in several studies [32, 33]. In our human study, we observed a significant increase in serum levels of CC-16 after pneumoperitoneum establishment. Hydromorphone exerted better anti-inflammatory and antioxidant effects than morphine during pneumoperitoneum, as shown by the PAB and ICAM-1 levels, which is consistent with other researchers' findings [35, 36]. Previous studies noted that the oxygen desaturation is below 95% in one-third of nonelderly adults receiving 2 mg intravenous hydromorphone [45]. However, in our clinical study, no serious respiratory decrease in oxygen desaturation was observed in patients. This may have been because some of the side effects of hydromorphone were covered under general anesthesia.

According to the relevant literature and the results of preliminary experiments, we developed a mouse model of pneumoperitoneum-induced lung injury [13, 46, 47]. After insufflation (1 h) and deflation (3 h) with CO_2_, we found leukocyte infiltration and visible alveolar structure damage in lung tissues by histological examination, which suggested the successful establishment of the mouse model [38, 48]. This was accompanied by cell damage as shown by increased LDH activity in BALF. Mitochondrial morphological and functional impairment during pneumoperitoneum has been shown to be involved in multiple organ injury [13, 49]. Herein, we found that during pneumoperitoneum, excessive pressure and prolonged exposure led to mitochondrial swelling, vacuolization, and fragment, which was consistent with previous studies [13, 50]. Multiple studies have demonstrated that mitochondrial DNA (mtDNA) is released after mitochondria are impaired [51], and mtDNA content has been shown to reflect the degree of mitochondrial dysfunction in response to cellular stress injury [51]. In addition, we measured the enzymatic activity of MPO in the mouse serum, an inflammatory biomarker that is highly enriched in azurophilic granules and released into the extracellular fluid upon inflammation [52]. In addition, TAS and TOS are sensitive indicators of the overall antioxidant and oxidation states, respectively [53]. The TOS/TAS ratio and oxidative stress index (OSI) are indicators of the degree of oxidative stress and provide a more accurate method of evaluating the changes in oxidant-antioxidant balance [53]. Thus, our findings showed that pretreatment with hydromorphone in mice subjected to pneumoperitoneum alleviated lung oxidative injury by increasing TAS while decreasing TOS, the OSI, and MPO activity and that these changes were accompanied by a reduction in mtDNA content and recovery of mitochondrial morphology to a certain extent. Thus, improvements of mitochondrial function may contribute to the antioxidant and anti-inflammatory effects of hydromorphone.

HO-1 in mitochondria plays a prominent role in different conditions such as ischemia-reperfusion, hypoxia, and sepsis resulting from oxidative dysregulation and inappropriate inflammatory responses [54, 55]. In addition, the protective properties of HO-1 are closely associated with mitochondrial dynamics [15, 56]. Our previous research proved that HO-1 exerts broad protective effects in LPS-activated RAW 264.7 cells and in rat models of endotoxin-induced lung injury by maintaining mitochondrial dynamics [15, 16]. In the current study, intraperitoneal CO_2_ insufflation shifted the balance toward a fission phenotype, and the levels of HO-1 were significantly increased in the lung tissues of mice subjected to pneumoperitoneum, which revealed that HO-1 exert endogenous protective effects in this context. To further verify that hydromorphone regulated HO-1-mediated mitochondrial dynamics, we established pneumoperitoneum in mice treated with HO-1-siRNA. Western blotting and immunofluorescence analysis confirmed that HO-1-siRNA was successfully delivered to mice. Hydromorphone inhibited mitochondrial fission induced by pneumoperitoneum, enhanced mitochondrial fusion, and further increased HO-1 expression in lung tissues. In contrast, HO-1 deficiency blocked the beneficial effects of hydromorphone on mitochondrial dynamics and increased mtDNA content in mice subjected to pneumoperitoneum. Collectively, these results suggested that HO-1-mediated mitochondrial dynamics contributes to the antioxidant and anti-inflammatory effects of hydromorphone against pneumoperitoneum-induced lung injury.

This study has several limitations. First, we did not perform in vitro experiments to more comprehensively elucidate the molecular mechanism by which hydromorphone protects against CO_2_ pneumoperitoneum-induced lung injury. Second, we could not collect lung tissues from patients because of ethical issues. Therefore, innovative research methods and further large-scale clinical research are required to determine the effects of hydromorphone on pulmonary function. Third, whether opioid receptors (*μ*, *δ*, *κ*) participate in the protective mechanism of hydromorphone against pneumoperitoneum-induced lung injury was not explored in this study. The underlying mechanisms of the indirect effects of opioid receptors need to be explored in the future.

**Figure 7:**
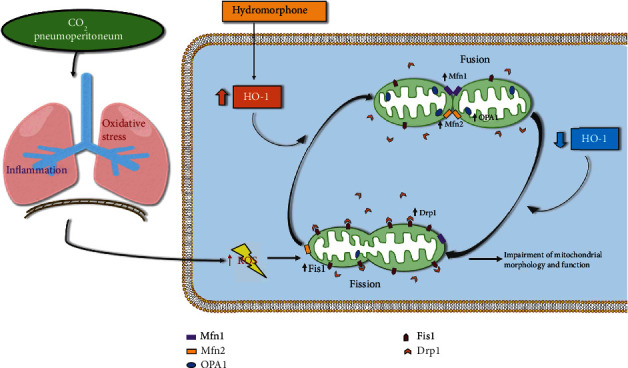
The protective mechanism of hydromorphone in CO_2_ pneumoperitoneum-induced lung injury. Pneumoperitoneum can induce oxidation and inflammatory damage in lung tissues. Excessive ROS production shifts the balance between mitochondrial fusion and fission in favor of increased fission, which impairs mitochondrial morphology and function. Hydromorphone pretreatment can increase the levels of HO-1 while preserving mitochondrial dynamics and improving mitochondrial function to protect against CO_2_ pneumoperitoneum-induced lung injury. ROS: reactive oxygen species; Mfn1: mitofusin 1; Mfn2: mitofusin 2; OPA1: optic atrophy type 1; Fis1: fission 1; Drp1: dynamin-related protein 1; HO-1: heme oxygenase-1.

In summary, the present study demonstrated that hydromorphone pretreatment increased the levels of HO-1 in animals as well as human patients during pneumoperitoneum and that HO-1 significantly blocked pneumoperitoneum-induced oxidative stress injury, strengthened the antioxidant defense system, alleviated inflammatory responses, and reduced the severity of pathological changes in the lung tissues, likely by preserving mitochondrial dynamics and improving mitochondrial function (Figure 7). The data obtained in our study provide significant new insights into the protective effects of hydromorphone against CO_2_ pneumoperitoneum-induced lung injury and offer ideas for new therapeutic strategies.

## Figures and Tables

**Table 1:** Patient demographics and characteristics.

Index	Hydromorphone (*n* = 25)	Morphine (*n* = 25)	*P* value
Age (years)	43.28 ± 10.24	44 ± 11.15	0.813
Weight (kg)	61.04 ± 6.31	63.96 ± 7.56	0.145
BMI (kg/m^2^)	23.48 ± 2.64	24.49 ± 3.5	0.258
Diagnosis, *n* (%)			0.863
Uterine myomas	13 (52%)	12 (48%)	
Ovarian cysts	9 (36%)	37 (44%)	
Endometrial carcinoma	2 (8%)	1 (4%)	
Cervical intraepithelial neoplasia	1 (4%)	0 (0%)	
Ovarian teratomas	0 (0%)	1 (4%)	
Duration of pneumoperitoneum (min)	145 (128, 167)	145 (130, 170)	0.876
Duration of anesthesia (min)	171 (160, 209)	180 (162, 199)	0.884
Recovery time (min)	20 (15, 26)	20 (15, 28)	0.914
Extubation time (min)	20 (15, 24)	20 (15, 25)	0.692
Total fluid intake (ml)	1500 (1500, 1600)	1500 (1425, 1800)	0.705
Bleeding volume (ml)	100 (80, 120)	100 (100, 175)	0.477
Urine volume (ml)	350 (275, 450)	300 (250, 425)	0.465

## Data Availability

The data used to support the findings of this study are available from the corresponding author upon request.

## Supplementary Materials

Supplementary Materials Supplementary Methods: immunofluorescence staining for neutrophils. Supplementary Figure 1: representative H&E stain sections and histological injury score of lung tissues under different doses of hydromorphone in mice subjected to pneumoperitoneum. Mice were pretreated with hydromorphone at different doses. Compared with group pretreatment with 2 mg/kg and 4 mg/kg, pretreatment with hydromorphone at 6 mg/kg in mice (aged 6~8 weeks, weighed 18~20 g) subjected to CO_2_ pneumoperitoneum was shown to be most effective in alleviating alveolar congestion and leukocyte infiltration of lung tissues. The lung injury scores at doses of 6 mg/kg were lower than those at doses of 2 mg/kg and 4 mg/kg in mice subjected to pneumoperitoneum. There was no statistical difference between the doses of 6 mg/kg and 8 mg/kg. Therefore, lower dose (6 mg/kg, 120 *μ*g) of hydromorphone was used. Red arrow indicated intra-alveolar hemorrhage, black arrows indicated inflammatory cell infiltration, green arrow indicated intra-alveolar congestion, and blue arrows indicated thickening of the alveolar wall. Original magnification, ×200, scale bar: 100 *μ*m. Lung injury scores are shown as mean ± SD. *P* values were calculated by one-way ANOVA corrected with Bonferroni coefficient (*n* = 6 per group). Supplementary Figure 2: representative immunofluorescence staining for DNA (DAPI, blue) and neutrophil (Ly6G^+^, green) and the percentage of neutrophils of merge images in each group. Neutrophil accumulation in lung tissues significantly increased in mice exposed to pneumoperitoneum and was significantly reduced following administration of the hydromorphone. But the effects were abolished in mice transfected with HO-1-siRNA. White arrows indicated neutrophil-specific Ly6G^+^ cells. Scale bar: 50 *μ*m. The data are the mean ± SD. *P* values were calculated by one-way ANOVA corrected with Bonferroni coefficient (*n* = 3 per group). Supplementary Figure 3: immunofluorescence staining for HO-1 in lung tissue sections of mice. Lung tissue sections were analyzed for HO-1 expression by immunofluorescence. In mice subjected to pneumoperitoneum, HO-1 expression was significantly intensive and widely distributed in lung tissues. HO-1 are stained green. Nuclei are stained blue. White arrows indicated positive staining areas of HO-1. Scale bar: 50 *μ*m. Supplementary Figure 4: representative images of TUNEL staining and measurements of TUNEL-positive cells in lung sections. The number of TUNEL-positive cells was significantly increased in mice transfected with HO-1-siRNA as compared to mice transfected with NC-siRNA. White arrows indicated TUNEL-positive cells. Scale bar: 100 *μ*m. The data are the mean ± SD. *P* values were calculated by independent-samples *t*-test (*n* = 6 per group).
