# Supplementary material for: Hydromorphone Protects against CO2 Pneumoperitoneum-Induced Lung Injury via Heme Oxygenase-1-Regulated Mitochondrial Dynamics
Source: Oxid Med Cell Longev. 2021 Apr 9;2021:9034376. doi: 10.1155/2021/9034376 (PMC8053056; doi:10.1155/2021/9034376)
Supplement: Supplementary Materials — Supplementary Methods: immunofluorescence staining for neutrophils. Supplementary Figure 1: representative H&E stain sections and histological injury score of lung tissues under different doses of hydromorphone in mice subjected to pneumoperitoneum. Mice were pretreated with hydromorphone at different doses. Compared with group pretreatment with 2 mg/kg and 4 mg/kg, pretreatment with hydromorphone at 6 mg/kg in mice (aged 6~8 weeks, weighed 18~20 g) subjected to CO2 pneumoperitoneum was shown to be most effective in alleviating alveolar congestion and leukocyte infiltration of lung tissues. The lung injury scores at doses of 6 mg/kg were lower than those at doses of 2 mg/kg and 4 mg/kg in mice subjected to pneumoperitoneum. There was no statistical difference between the doses of 6 mg/kg and 8 mg/kg. Therefore, lower dose (6 mg/kg, 120 μg) of hydromorphone was used. Red arrow indicated intra-alveolar hemorrhage, black arrows indicated inflammatory cell infiltration, green arrow indicated intra-alveolar congestion, and blue arrows indicated thickening of the alveolar wall. Original magnification, ×200, scale bar: 100 μm. Lung injury scores are shown as mean ± SD. P values were calculated by one-way ANOVA corrected with Bonferroni coefficient (n = 6 per group). Supplementary Figure 2: representative immunofluorescence staining for DNA (DAPI, blue) and neutrophil (Ly6G+, green) and the percentage of neutrophils of merge images in each group. Neutrophil accumulation in lung tissues significantly increased in mice exposed to pneumoperitoneum and was significantly reduced following administration of the hydromorphone. But the effects were abolished in mice transfected with HO-1-siRNA. White arrows indicated neutrophil-specific Ly6G+ cells. Scale bar: 50 μm. The data are the mean ± SD. P values were calculated by one-way ANOVA corrected with Bonferroni coefficient (n = 3 per group). Supplementary Figure 3: immunofluorescence staining for HO-1 in lung tissue sections of mic [file 9034376.f1.doc]

**Supplementary Materials**

**Hydromorphone** **Protects** **Against** **CO_2_** **Pneumoperitoneum- Induced Lung Injury via** **Heme Oxygenase-1-****Regulated** **Mitochondrial Dynamics**

Jia Shi, ^1^ Shi-Han Du, ^1^ Jian-Bo Yu ^1^, Yan-Fang Zhang, ^1^ Si-Meng He, ^2^ Shu-An Dong,^1^ Yuan Zhang, ^1^ Li-Li Wu, ^1^ Cui Li, ^1^ and Hai-Bo Li ^1^

*^1^* *Department of* *Anesthesiology and Critical Care Medicine,* *Tianjin Nankai Hospital, Tianjin Medical University, Tianjin 300100, China*

*^2^ Department of Anesthesiology and Critical Care Medicine,* *Tianjin Nankai Hospital, NanKai University, Tianjin 300100, China*

Correspondence should be addressed to Jian-Bo Yu; [yujianbo11@126.com](mailto:yujianbo11@126.com)

Jia Shi, Shi-Han Du and Jian-Bo Yu contributed equally to this article.

**Supplementary Methods**

**Immunofluorescent staining for neutrophils**

The paraffin-embedded lung sections were used for immunofluorescent staining for neutrophils. The Ly6G antibody (1:200, #ab238132, Abcam, USA) and the ﬂuorescent-labeled secondary antibodies (1:400, Alexa Fluor 594, BioLegend, USA) were used to incubate the sections. Then the Ly6G^+^ cells were observed under an Olympus ﬂuorescence microscope. Percentages of the positive area were calculated in relation to the whole visual field with the ImageJ Software.

**
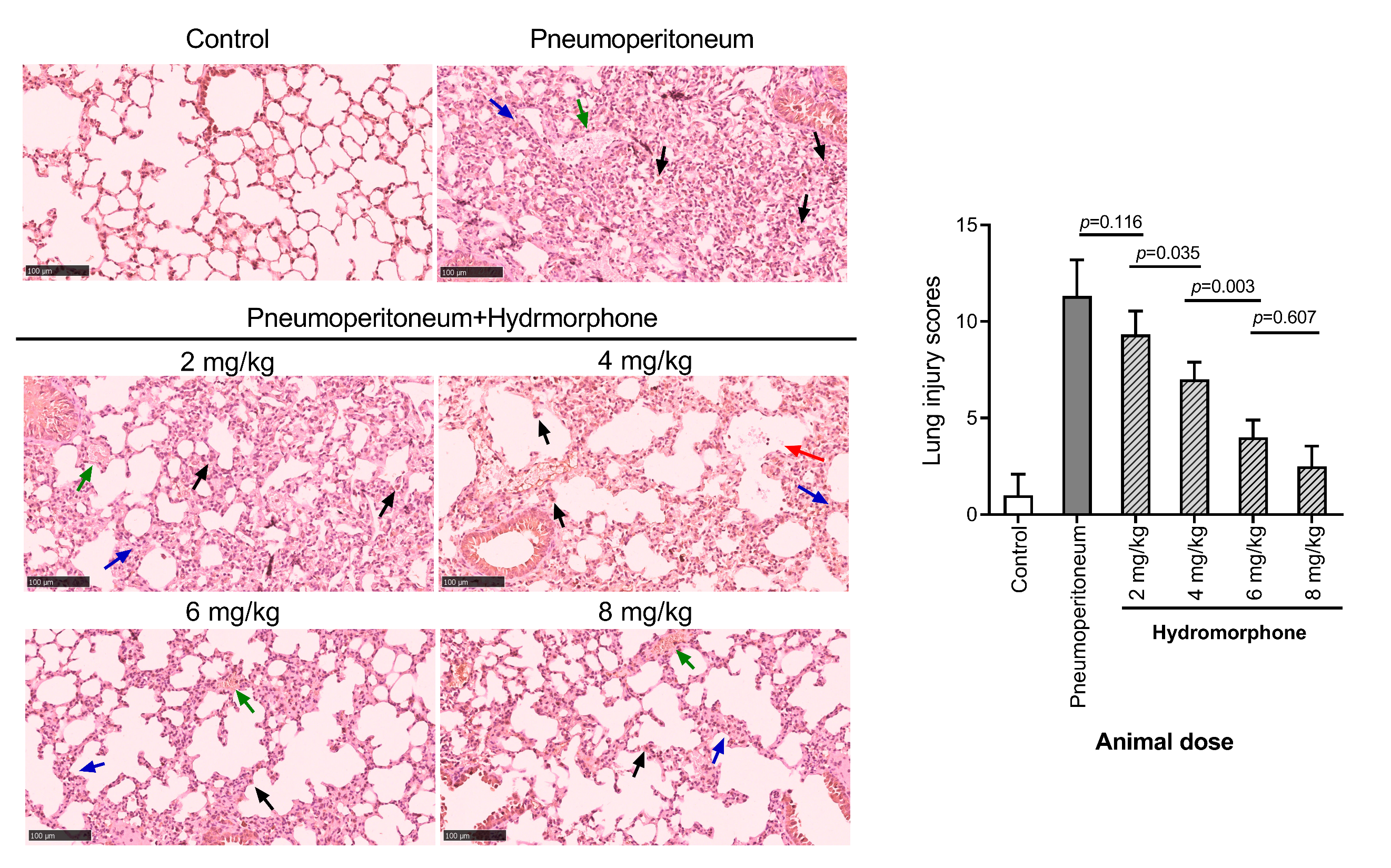
**

**Supplementary Fig. S1.** Representative H&E stain sections and histological injury score of lung tissues under different doses of hydromorphone in mice subjected to pneumoperitoneum. Mice were pre-treatment with hydromorphone at different doses. Compared with group pre-treatment with 2 mg/kg and 4 mg/kg, pre-treatment with hydromorphone at 6 mg/kg in mice (aged 6~8 weeks, weighed 18~20 g) subjected to CO_2_ pneumoperitoneum were shown to be most effective in alleviating alveolar congestion and leukocyte infiltration of lung tissues. The lung injury scores at doses of 6 mg/kg were lower than 2 mg/kg and 4 mg/kg in mice subjected to pneumoperitoneum. There was no statistical difference between the dose of 6 mg/kg and 8 mg/kg. Therefore, a lower dose (6 mg/kg, 120 *μ*g) of hydromorphone was used. Red arrow indicated intra-alveolar hemorrhage, black arrows indicated inflammatory cell infiltration, green arrow indicated intra-alveolar congestion and blue arrows indicated thickening of the alveolar wall. Original magnification, 200 ×, Scale bar: 100 *μ*m. Lung injury scores are shown as mean ± SD. *P* values were calculated by the one-way ANOVA corrected with Bonferroni coefficient (n=6 per group).

**
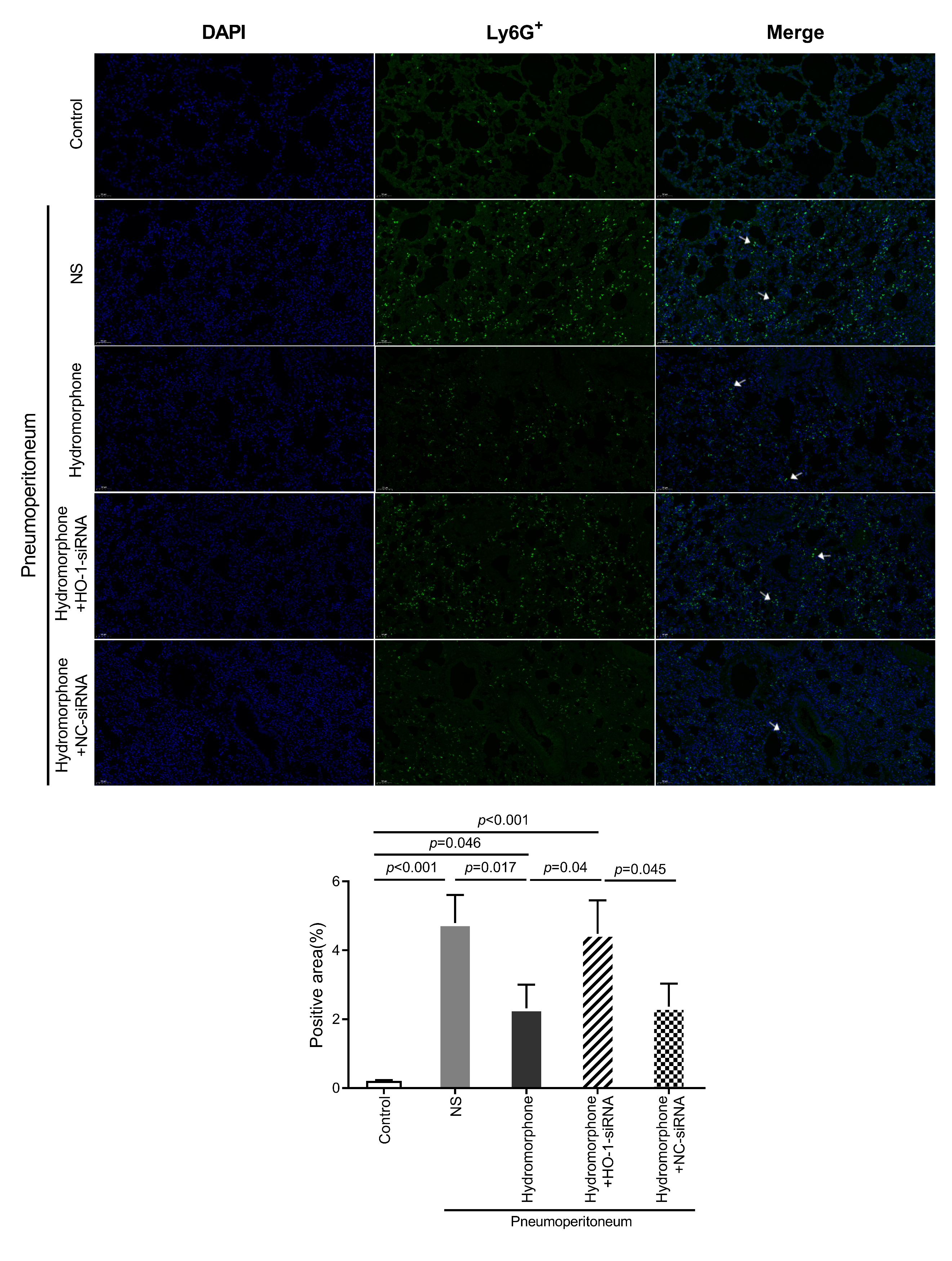
**

**Supplementary Fig. S2.** Representative immunofluorescent staining for DNA (DAPI, blue) and neutrophil (Ly6G^+^, green) and the percentage of neutrophils of merge images in each group. Neutrophil accumulation in lung tissues significantly increased in mice exposed to pneumoperitoneum and was significantly reduced following administration of the hydromorphone. But the effects were abolished in mice transfected with HO-1-siRNA. White arrows indicated neutrophil-specific Ly6G^+^ cells. Scale bar: 50 *μ*m. The data are the mean ± SD. *P* values were calculated by one-way ANOVA corrected with Bonferroni coefficient (n=3 per group).


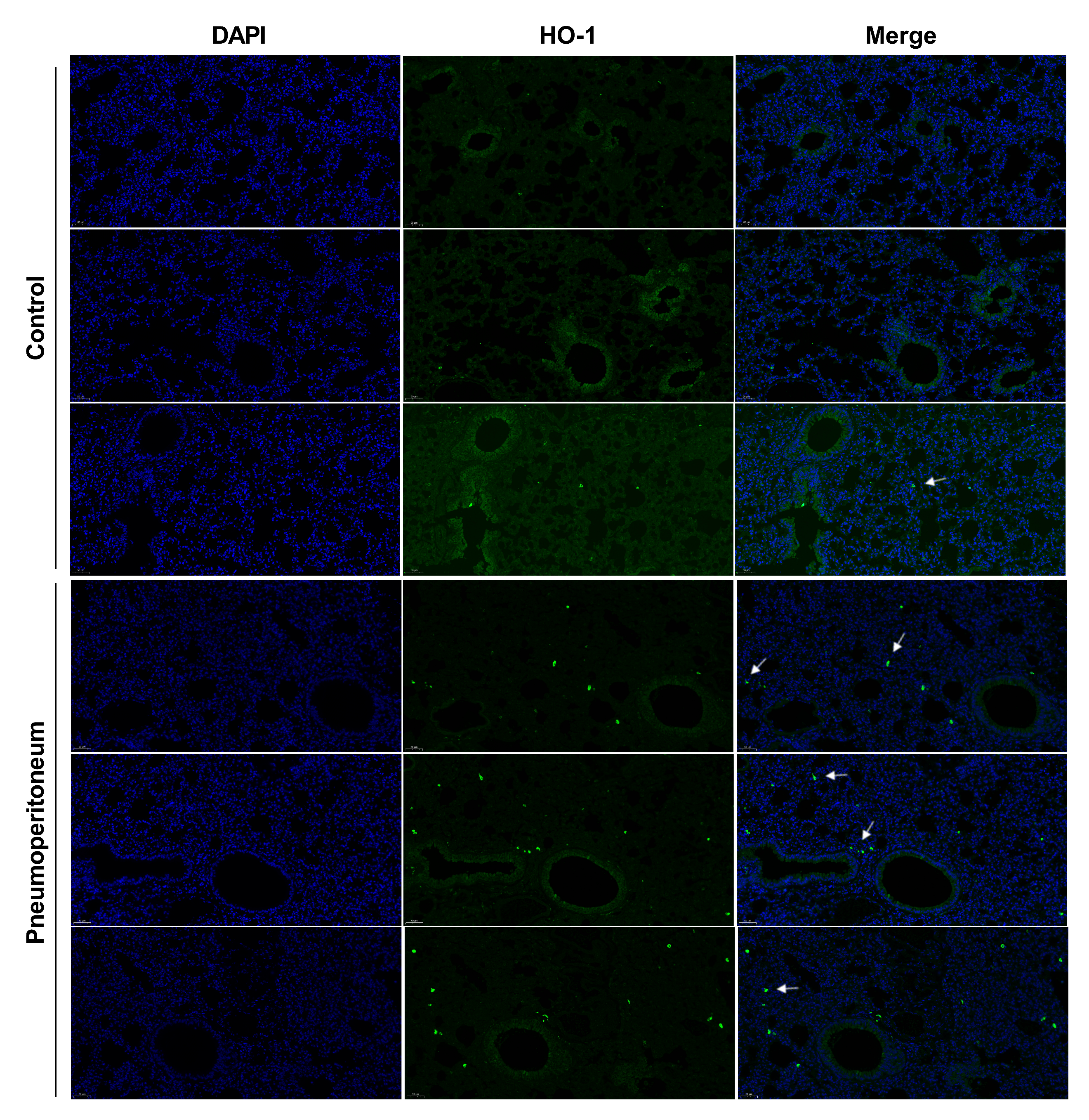


**Supplementary Fig. S3.** Immunofluorescence staining for HO-1 in lung tissue sections of mice. Lung tissue sections were analyzed for HO-1 expression by immunofluorescence. In mice subjected to pneumoperitoneum, HO-1 expression was significantly intensive and widely distributed in lung tissues. HO-1 are stained green. Nuclei are stained blue. White arrows indicated positive staining areas of HO-1. Scale bar: 50 *μ*m. **
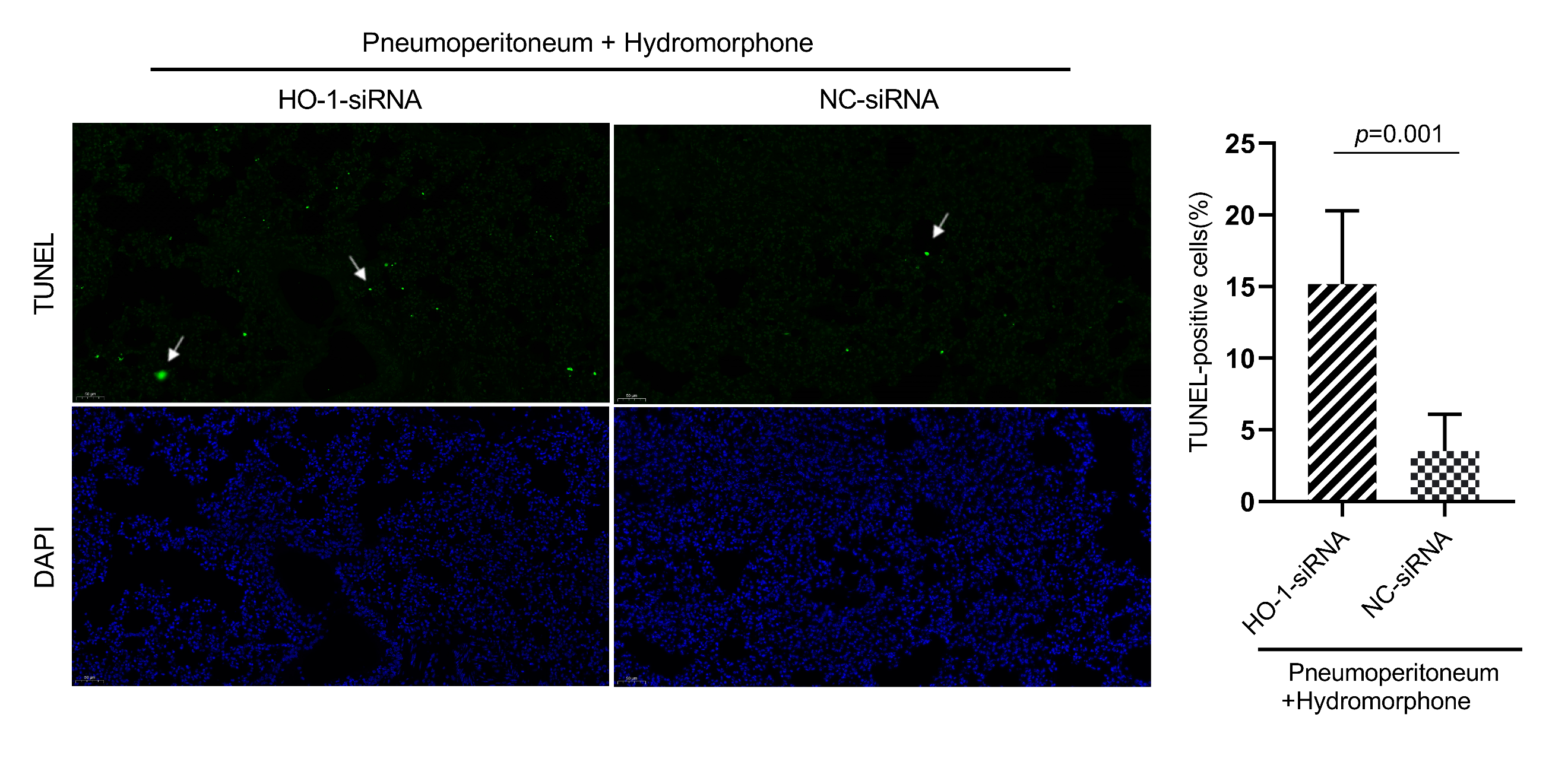
**

**Supplementary Fig. S4.** Representative images of TUNEL staining and measurements of TUNEL-positive cells in lung sections. The number of TUNEL-positive cells was significantly increased in mice transfected with HO-1-siRNA as compare to mice transfected with NC-siRNA. White arrows indicated Tunnel positive cells. Scale bar: 100 *μ*m. The data are the mean ± SD. *P* values were calculated by independent-samples *t*-test (n=6 per group).
